# Tailored Bayes: a risk modeling framework under unequal misclassification costs

**DOI:** 10.1093/biostatistics/kxab023

**Published:** 2021-08-07

**Authors:** Solon Karapanagiotis, Umberto Benedetto, Sach Mukherjee, Paul D W Kirk, Paul J Newcombe

**Affiliations:** MRC Biostatistics Unit, University of Cambridge, UK and The Alan Turing Institute, UK; Bristol Heart Institute, University of Bristol, UK; German Center for Neurodegenerative Diseases (DZNE), Bonn, Germany and MRC Biostatistics Unit, University of Cambridge, UK; MRC Biostatistics Unit, University of Cambridge, UK; MRC Biostatistics Unit, University of Cambridge, UK

**Keywords:** Bayesian inference, Binary classification, Misclassification costs, Tailored Bayesian methods

## Abstract

Risk prediction models are a crucial tool in healthcare. Risk prediction models with a binary outcome (i.e., binary classification models) are often constructed using methodology which assumes the costs of different classification errors are equal. In many healthcare applications, this assumption is not valid, and the differences between misclassification costs can be quite large. For instance, in a diagnostic setting, the cost of misdiagnosing a person with a life-threatening disease as healthy may be larger than the cost of misdiagnosing a healthy person as a patient. In this article, we present Tailored Bayes (TB), a novel Bayesian inference framework which “tailors” model fitting to optimize predictive performance with respect to unbalanced misclassification costs. We use simulation studies to showcase when TB is expected to outperform standard Bayesian methods in the context of logistic regression. We then apply TB to three real-world applications, a cardiac surgery, a breast cancer prognostication task, and a breast cancer tumor classification task and demonstrate the improvement in predictive performance over standard methods.

## 1. Introduction

Risk prediction models are widely used in healthcare (Roques *and others*, 2003; Hippisley-Cox *and others*, 2008; Wishart *and others*, 2012). In both diagnostic and prognostic settings, risk prediction models are regularly developed, validated, implemented, and updated with the aim of assisting clinicians and individuals in estimating probabilities of outcomes of interest which may ultimately guide their decision making (Down *and others*, 2014; NICE, 2016; Baumgartner *and others*, 2017). The most common type of risk prediction model is based on binary outcomes, with class labels 0 (negative) and 1 (positive). Models for binary outcomes are often constructed to minimize the expected classification error; that is, the proportion of incorrect classifications (Zhang, 2004; Steinwart, 2005; Bartlett *and others*, 2006). We refer to this paradigm as the standard classification paradigm. The disadvantage of this paradigm is that it implicitly assumes that all classification errors have equal costs, that is, the cost of misclassification of a positive label equals the cost of misclassification of a negative label. (Throughout the document, we refer to the costs of incorrect classifications as misclassification costs). However, equal costs may not always be appropriate and will depend on the scientific or medical context. For example, in cancer diagnosis, a false negative (i.e., misdiagnosing a cancer patient as healthy) could have more severe consequences than a false positive (i.e., misdiagnosing a healthy individual with cancer); the latter may lead to extra medical costs and unnecessary anxiety for the individual but not result in loss of life.^1^ For such applications, a prioritized control of asymmetric misclassification costs is needed.

To meet this need, different methods have been developed. In the machine learning literature, they are studied under the term cost-sensitive learning (Elkan, 2001). Existing research on cost-sensitive learning can be grouped into two main categories: direct and indirect approaches. Direct approaches aim to make particular classification algorithms cost-sensitive by incorporating different misclassification costs into the training process. This amounts to changing the objective/likelihood function that is optimized when training the model (e.g., Kukar *and others*, 1998; Ling *and others*, 2004; Masnadi-Shirazi and Vasconcelos, 2010). A limitation is that these approaches are designed to be problem-specific, requiring considerable knowledge of the model in conjunction with its theoretical properties, and possibly new computational tools. Conversely, indirect approaches are more general because they achieve cost-sensitivity without any, or with minor modification to existing modeling frameworks. In this article, we focus on indirect approaches.

Indirect methods can be further subdivided into thresholding and sampling/weighting. Thresholding is the simplest approach of the two, as it changes the classification threshold of an existing risk prediction model. We can use the threshold to classify datapoints into positive or negative status if the model can produce probability estimates. This strategy is optimal if the true class probabilities were available. In other words, if the model is based on the logarithm of the ratio of true class probabilities, the threshold should be modified by a value equal to the logarithm of the ratio of misclassification costs (Duda *and others*, 2012). This is based on decision theoretic arguments as we show in Section 2 (Pauker and Kassirer, 1975; Duda *and others*, 2012). In practice, however, this strategy may lead to sub-optimal solutions. We demonstrate this using synthetic (Section 3) and real-life data (Section 4).

Alternatively, sampling methods modify the distribution of the training data according to misclassification costs (see Elkan (2001) for a theoretical justification). This can be achieved by generating new datapoints from the class with smaller numbers of datapoints, that is, oversampling from the minority class, or by removing datapoints from the majority class (undersampling). The simplest form is random sampling (over- or under-). However, both come with drawbacks. Duplicating samples from the minority class may cause overfitting (Zadrozny *and others*, 2003). Similarly, random elimination of samples from the majority class can result in loss of data which might be useful for the learning process. Weighting (e.g., Ting, 1998; Margineantu and Dietterich, 2003) can also be conceptually viewed as a sampling method, where weights are assigned proportionally to misclassification costs. For example, datapoints of the minority class, which usually carries a higher misclassification cost, may be assigned higher weights. Datapoints with high weights can be viewed as sample duplication – thus oversampling. In general, random sampling/weighting determine the datapoints to be duplicated or eliminated based on outcome information (whether a datapoint belongs to the majority or the minority class). Notably, they do not take into account critical regions of the covariate space, such as regions that are closer to the target decision boundary. A decision boundary specifies distinct classification regions on the covariate space based on specified misclassification costs (see Section 3 for details). This is the goal of the framework presented here.

In this article, we build upon the seminal work of Hand and Vinciotti (2003), and present an umbrella framework that allows us to incorporate misclassification costs into commonly used models for binary outcomes. The framework allows us to tailor model development with the aim of improving performance in the presence of unequal misclassification costs. Although the concepts we discuss are general, and allow for relatively simple tailoring of a wide range of models (essentially whenever the objective function can be expressed as a sum over samples), we focus on a Bayesian regression paradigm. Hence, we present Tailored Bayes (TB), a framework for tailored Bayesian inference when different classification errors incur different penalties. We use a decision theoretic approach to quantify the benefits and costs of correct and incorrect classifications (Section 2). The method is based on the principle that the relative harms of false positives and false negatives can be expressed in terms of a target threshold. We then build a 2-stage model (Section 2.3); first introduced by Hand and Vinciotti (2003). In the first stage, the most informative datapoints are identified. A datapoint is treated as informative if it is close to the target threshold of interest. Each datapoint is assigned a weight proportional to its distance from the target threshold. Intuitively, one would expect improvements in performance to be possible by putting decreasing weights on the class labels of the successively more distant datapoints. In the second stage, these weights are used to downweight each datapoint’s likelihood contribution during model training. A key feature is that this changes the estimation output in a way that goes beyond thresholding and we demonstrate this effect in simple examples (Section 3).

We conduct simulation studies to illustrate the improvement in predictive performance of our proposed TB modeling framework over the standard Bayesian paradigm (Section 3). We then apply the methodology to three real-data applications (Section 4). Our two main case studies are a breast cancer and a cardiac surgery prognostication task where we have information on how clinicians prioritize different classification errors. We show that incorporating this information into the model through our TB approach leads to better treatment decisions. We finish with a discussion of our approach, findings and provide some general remarks in Section 5.

## 2. Methods

We use a decision theoretic approach to summarize the costs of misclassifications of a binary outcome into a single number, which we refer to as the target threshold (Section 2.1). We later (Section 2.2) define the expected utility of risk prediction and use the target threshold and the never treat policy to simplify the expected utility and derive the Net Benefit of a risk prediction model. We use the Net Benefit as our model evaluation metric throughout the article. In Section 2.3, we incorporate the target threshold in the model formulation which results in the tailored likelihood function (Section 2.4) and the tailored posterior (Section 2.5).

### 2.1. The target threshold

Let $Y \in \{0, 1\}$ represent a binary outcome of interest. The observed $Y$ is a realization of a binary random variable following a Bernoulli distribution with $\pi = P[Y = 1]$. This is the marginal probability of the outcome being present, and consequently, the probability the outcome being absent is $(1 - \pi)$.

We introduce utility functions to take into account the benefits or harms of different classifications. A utility function assigns a value to each of the four possible classification-outcome combinations stating exactly how beneficial/costly each action (treat or no treat) is. We assume that people who are classified as positive receive treatment and people who are classified as negative do not receive treatment. We use “treatment” in the generic sense of healthcare intervention which could be a drug, surgery, or further testing. Each possible combination of classification (negative and positive) and outcome status (0, 1) is associated with an utility where a positive value indicates a benefit and a negative value indicates a cost or harm. The four utilities associated with binary classification problems are: (i) $U_{\rm TP}$, the utility of a true positive classification, that is administering treatment to a patient who has the outcome (i.e., treat when necessary), (ii) $U_{\rm FP}$, the utility of a false positive classification, that is the utility of administering treatment to a patient who does not have the outcome (i.e., administering unnecessary treatment), (iii) $U_{\rm FN}$, the utility of a false negative classification, that is the utility of withholding treatment from a patient that has the outcome (i.e., withholding beneficial treatment), and (iv) $U_{\rm TN}$, the utility of a true negative classification, that is the utility of withholding treatment from a patient who does not have the outcome (i.e., withholding unnecessary treatment).

The expected utilities of the two fixed courses of action (or policies) of always treat and never treat are given by

(2.1a) \begin{eqnarray*} \label{EU_treat} {\rm EU}_{\rm treat} &=& \pi U_{\rm TP} + (1 - \pi) U_{\rm FP},\\ \end{eqnarray*}

(2.1b) \begin{eqnarray*} {\rm EU}_{\rm no \text{ } treat} &=& \pi U_{\rm FN} + (1 - \pi) U_{\rm TN},\label{EU_notreat} \end{eqnarray*}

where ${\rm EU}_{\rm treat}$ and ${\rm EU}_{\rm no \text{ } treat}$ are the expected utility of treating and not treating, respectively. In principle, one should choose the course of action with the highest expected utility. When the expected utilities are equal, the decision maker is indifferent on the course of action (Pauker and Kassirer, 1975). Based on classical decision theory, we employ the threshold concept and denote with $t$ the threshold at which the decision maker is indifferent on the course of action (Pauker and Kassirer, 1980). This is the principle of clinical equipoise which exists when all of the available evidence about a course of action does not show that it is more beneficial than an alternative and, equally, does not show that it is less beneficial than the alternative (Turner, 2013). Clinical equipoise is regarded as an “ethically necessary condition in all cases of clinical research” (Freedman, 1987). Based on the threshold concept, an individual should be treated (i.e., classified as positive) if $\pi \geq t$ and should not be treated (i.e., classified as negative) otherwise. Having defined $t$ as the value of $\pi$ of clinical equipoise where the expected benefit of treatment is equal to the expected benefit of avoiding treatment implies ${\rm EU}_{\rm treat} = {\rm EU}_{\rm no \text{ } treat}$ or equivalently, $t U_{\rm TP} + (1 - t) U_{\rm FP} = t U_{\rm FN} + (1 - t) U_{\rm TN}$. Solving for $t$,

(2.2) \begin{equation*} \begin{split} t = \frac{U_{\rm TN} - U_{\rm FP}}{U_{\rm TN} - U_{\rm FP} + U_{\rm TP} - U_{\rm FN}}\\ = \frac{H}{H + B} = \frac{1}{1 + \frac{B}{H}}, \label{target_threshold} \end{split} \end{equation*}

where $B = U_{\rm TP} - U_{\rm FN}$ is the difference between the utility of administering treatment to individuals who have the outcome and the utility of withholding treatment in those who have the outcome. In other words, $B$ is the benefit for positive prediction, and consequent treatment, among those with the outcome. Similarly, $B$ can be interpreted as the consequence of failing to treat when it would have been of benefit, that is, the harm from a false negative result (compared to a true positive result). Comparably, $H$ is the difference between the utility of avoiding treatment in patients who do not have the outcome and the utility of administering treatment to those who do not have the outcome (i.e., $U_{\rm TN} - U_{\rm FP}$). In other words, $H$ is the consequence of being treated unnecessarily, this is the harm associated with a false positive result (compared to a true negative result).

We henceforth refer to $t$ as the target threshold. Alternative names in the literature are risk threshold (Baker *and others*, 2009) and threshold probability (Tsalatsanis *and others*, 2010). It is a scalar function of $U_{\rm TP},U_{\rm FN}, U_{\rm TN}$, and $U_{\rm FP}$ that determines the cut-off point for calling a result positive that maximizes expected utility. Equation (2.2) therefore tells us that the target threshold at which the decision maker will opt for treatment is informative of how they weigh the relative harms of false positive and false negative results. The main advantage of this decision theoretic approach is there is no need to explicitly specify the relevant utilities, but only the desired target threshold.

**Example:** Assume that for every correctly treated patient (true positive) we are willing to incorrectly treat 9 healthy individuals (false positives).^2^ Then, we consider the benefit of correctly treating a patient to be nine times larger than the harm of an unnecessary treatment: the harm-to-benefit ratio is 1:9. This ratio has a direct relationship to $t$: the odds of $t$ equal the harm-to-benefit ratio. That is, $H/B = t / (1 - t)$ which is implied by (2.2). For example, $t$ of 10% implies a harm-to-benefit ratio of 1:9 (odds(10%) = 10/90).

### 2.2. Net benefit for risk prediction

In practice, we do not know the probability of the outcome of any given individual. Instead, we need to estimate it, according to a set of covariates. Let $\mathbf{X} \in \mathbb{R}^d$ be a vector of $d$ covariates and define $\pi(\mathbf{x})$ as the conditional class 1 probability given the observed values of the covariates, $\mathbf{x}: \pi(\mathbf{x}) = P[Y = 1 \mid \mathbf{X} = \mathbf{x}]$. We are concerned with the problem of classifying future values of $Y$ from the information that the covariates $\mathbf{X}$ contain. Assume we have a prediction model and an estimate of $\pi(\mathbf{x})$, denoted $\hat{\pi}(\mathbf{x})$. We classify an individual as positive if $\hat{\pi}(\mathbf{x}) \ge t$, where $t$ is the target threshold (defined in (2.2)) and as negative otherwise. The expected utility of assigning treatment or not (i.e., classifying positive or negative) at $t$ based on the model’s predictions $\hat{\pi}(\mathbf{x})$ can be written as

(2.3) \begin{equation*} \begin{split} {\rm EU}_{{\rm Pred}(t)} = P(\hat{\pi}(\mathbf{x}) \geq t, y = 1) U_{\rm TP} + P(\hat{\pi}(\mathbf{x}) < t, y = 1) U_{\rm FN} + \\ P(\hat{\pi}(\mathbf{x}) < t, y = 0) U_{\rm TN} + P(\hat{\pi}(\mathbf{x}) \geq t, y = 0) U_{\rm FP} \\ = \pi {\rm TPR}_t U_{\rm TP} + \pi (1 - {\rm TPR}_t) U_{\rm FN} + (1 - \pi) {\rm FPR}_t U_{\rm FP} + (1 - \pi) (1-{\rm FPR}_t) U_{\rm TN}\\ = \{\pi {\rm TPR}_t B - (1 - \pi) {\rm FPR}_t H \} + \{\pi U_{\rm FN} + (1 - \pi) U_{\rm TN} \}, \end{split} \label{full_EU} \end{equation*}

where ${\rm TPR}_t$ is the true positive rate, that is, $P(\hat{\pi}(\mathbf{x}) \geq t|y = 1)$ and ${\rm FPR}_t$ is the false positive rate, that is, $P(\hat{\pi}(\mathbf{x}) \geq t|y = 0)$. The drawback of this formulation is the need to specify the four utilities. Equation (2.3) can be simplified by considering the expected utility of risk prediction in excess of the expected utility of no treatment. The expected utility of no treatment is given in (2.1b), and so, subtracting this from both sides of (2.3), the expected utility of risk prediction in excess of the expected utility of no treatment is

(2.4) \begin{equation*} \begin{split} {\rm EU}_{{\rm Pred}(t)} - {\rm EU}_{\rm no \text{ } treat} = \pi {\rm TPR}_t B - (1 - \pi) {\rm FPR}_t H\\ = B \big\{ \pi {\rm TPR}_t - (1 - \pi) {\rm FPR}_t \frac{t}{1 - t}\big\}.\end{split} \label{net_benefit_long} \end{equation*}

This is a Hippocratic utility function because it is motivated by the Hippocratic oath; do the best in ones ability (beneficence) and do no harm (nonmaleficence) (Childress and Beauchamp, 2001). To be consistent with the Hippocratic oath, the modeler chooses the model that has the greatest chance of giving an outcome no worse than the outcome of no treatment. With $B = 1$, (2.4) is defined as the Net Benefit of risk prediction versus treat none (Vickers and Elkin, 2006; Baker *and others*, 2009). Setting $B = 1$ as the reference level means that Net Benefit is measured in units of true positive predictions. To see this we re-write (2.4) as

(2.5) \begin{equation*} {\rm NB}_{{\rm Pred}(t)} = \frac{{\rm TP}_t}{n} - \frac{{\rm FP}_t}{n} \frac{t}{1 - t}, \label{NB_model} \end{equation*}

where $\mathrm{TP}_t$ is number of patients with true positive results, $\mathrm{FP}_t$ is number of patients with false positive results, and $n$ is the sample size. To simplify notation we write NB instead of $\mathrm{NB}_{\mathrm{Pred}(t)}$. NB gives the proportion of net true positives in the data set, accounting for the different misclassification costs. In other words, the observed number of true positives is corrected for the observed proportion of false positives weighted by the odds of the target threshold, and the result is divided by the sample size. This net proportion is equivalent to the proportion of true positives in the absence of false positives. For instance, a NB of 0.05 for a given target threshold, can be interpreted as meaning that use of the model, as opposed to simply assuming that all patients are negative, leads to the equivalent of an additional 5 net true positives per 100 patients.

For the remainder of the manuscript NB will be our main performance measure for model evaluation. We have written NB as a function of the target threshold $t$, which allows information about the relative utilities of treatments to be included in our model formulation, which we now show.

### 2.3. Model formulation

Denote data $D = \{ (y_i, \mathbf{x}_i) :i= 1, \dots, n \}$ where $y_i$ is the outcome indicating the class to which the $i^{th}$ datapoint belongs and $\mathbf{x}_i$ is the vector of covariates of size $d$. The objective is to estimate the posterior probability of belonging to one of the classes given a set of new datapoints. We use $D$ to fit a model $p(y_i \mid \mathbf{x}_i)$ and use it to obtain $\pi(\mathbf{x}_*)$ for a future datapoint $y_*$ with covariates $\mathbf{x}_*$. We simplify the structure using $p(y_i \mid f(\mathbf{x}_i))$, where $f: \mathcal{X} \to \mathbb{R}$ is a function that maps the vector of the covariates to the real line i.e., the linear predictor used in generalized linear models. To develop the complete model, we need to specify $p(y_i \mid f(\mathbf{x}_i))$ and $f$.

In the machine learning literature, most of the binary classification procedures use a loss-function-based approach. In the same spirit, we model $p(y_i \mid f(\mathbf{x}_i))$ according to a loss function $\ell(y_i, f(\mathbf{x}_i))$ which measures the loss for reporting $f$ when the truth is $y$. Mathematically, minimizing this loss function can be equivalent to maximizing $-\ell(y, f)$, where $\exp \{ -\ell(y, f)\}$ is proportional to the likelihood function. This duality between “likelihood” and “loss,” that is viewing the loss as the negative of the log-likelihood is referred to in the Bayesian literature as a logarithmic score (or loss) function (Bernardo and Smith, 2009; Bissiri *and others*, 2016). A few popular choices of loss functions for binary classification are the exponential loss used in boosting classifiers (Friedman *and others*, 2000), the hinge loss of support vector machines (Zhang, 2004), or logistic loss of logistic regression (Friedman *and others*, 2000; Zhang, 2004). In this article, we focus on the following loss,

(2.6) \begin{equation*} \ell_{w_i}(y_i, f(\mathbf{x}_i)) = -\pi(f(\mathbf{x}_i))^{w_{i}y_i} (1- \pi(f(\mathbf{x}_i)))^{w_i(1 - y_i)}, \text{ for } i = 1, \ldots, n \label{hand_loss} \end{equation*}

where we define $\pi_{w_{i}}(f(\mathbf{x}_i)) := \pi(f(\mathbf{x}_i))^{w_i} =(\exp \{ {\mathbf{x}^T_i \boldsymbol{\beta}} \}/ 1 + \exp\{ {\mathbf{x}^T_i \boldsymbol{\beta}}\})^{w_i}$ and $w_i \in[0,1]$ are datapoint-specific weights. This is a generalized version of the logistic loss, first introduced by Hand and Vinciotti (2003). We recover the standard logistic loss by setting $w_i = 1$ for all $i = 1, \dots,n$. Note that we specify $f$ as a linear function, i.e., $f(\mathbf{x}_i)= \mathbf{x}^T_i \boldsymbol{\beta}$, where $\boldsymbol{\beta}$ is a $d + 1$ dimensional vector of regression coefficients. Hence, our objective is to learn $\boldsymbol{\beta}$. We make this explicit by replacing $\pi_{w_{i}}(f(\mathbf{x}_i))$ with $\pi_{w_{i}}(\mathbf{x}_i;\boldsymbol{\beta})$ for the rest of this work.

The datapoint-specific weights, $w_i$, allow us to tailor the standard logistic model. We wish to weigh observations based on their vicinity to the target threshold, $t$, upweighting observations close to $t$ (the most informative) and downweighting those that are further away. To accomplish this, we set the weights as

(2.7) \begin{equation*} w_i = \exp \big \{-\lambda h(\pi_u(\mathbf{x}_i), t) \big \} = \exp \big \{-\lambda (\pi_u(\mathbf{x}_i) - t)^2 \big \}, \label{weights} \end{equation*}

where $h$ is the squared distance (see Supplementary material available at *Biostatistics* online for other options) and $\pi_u(\mathbf{x}_i)$ is the unweighted version of $\pi_{w_{i}}(\mathbf{x}_i;\boldsymbol{\beta})$. Of course, in practice we do not know $\pi_u(\mathbf{x}_i)$ so we cannot measure the distance between $t$ and each datapoint’s predicted probability, $\pi_u(\mathbf{x}_i)$, in order to derive these weights. To overcome this, we propose a two-stage procedure. First, the distance is measured according to an estimate of $\pi_u(\mathbf{x}_i)$, $\hat{\pi}_u(\mathbf{x}_i)$, which can be compared with $t$ to yield the weights. This estimate could be based on any classification method: we use standard unweighted Bayesian logistic regression in the analysis below. If a well-established model of $\pi_u(\mathbf{x}_i)$ already exists in the literature that could be used (as in our cardiac surgery case study, see Section 4.2) this task would not be necessary. After deriving the weights, they are then used to estimate $\pi_{w_{i}}(\mathbf{x}_i;\boldsymbol{\beta})$. Finally, under the formulation in (2.7) the weights decrease with increasing distance from the target threshold $t$. The tuning parameter $\lambda \geq 0$ controls the rate of that decrease. For $\lambda = 0$ we recover the standard logistic regression model. We use cross-validation to choose $\lambda$, see later for details.

### 2.4. Tailored likelihood function

To gain a better insight into the model, we define the tailored likelihood function as

(2.8) \begin{equation*} L(D \mid \boldsymbol{\beta}) = -\prod_{i=1}^{n} \ell_{w_i}(y_i, \mathbf{x}^T_i \boldsymbol{\beta}) = \prod_{i=1}^{n} \Bigg( \frac{\exp \{ {\mathbf{x}^T_i \boldsymbol{\beta}}\}}{1 + \exp \{ {\mathbf{x}^T_i \boldsymbol{\beta}\}}} \Bigg)^{y_iw_i} \Bigg( 1 - \frac{\exp \{ {\mathbf{x}^T_i \boldsymbol{\beta}}\}}{1 + \exp \{ {\mathbf{x}^T_i \boldsymbol{\beta}}\}} \Bigg)^{w_i (1-y_i)}.\label{tailored_likelihood} \end{equation*}

Strictly speaking, this quantity is not the standard logistic likelihood function. Nevertheless, it is instinctive to see its correspondence with the standard likelihood function. Thus, we rewrite (2.8) (after taking the log in both sides) as

(2.9) \begin{align*} \log(L(D \mid \boldsymbol{\beta})) &= -\sum_{i=1}^{n} \log(\ell_{w_i}(y_i, \mathbf{x}^T_i \boldsymbol{\beta}))\notag\\ &= \sum_{i=1}^{n} {y_iw_i} \log\Bigg( \frac{\exp \{ {\mathbf{x}^T_i \boldsymbol{\beta}}\}}{1 + \exp \{ {\mathbf{x}^T_i \boldsymbol{\beta}\}}} \Bigg) + {w_i (1-y_i)\log \Bigg( 1 - \frac{\exp \{ {\mathbf{x}^T_i \boldsymbol{\beta}}\}}{1 + \exp \{ {\mathbf{x}^T_i \boldsymbol{\beta}}\}} \Bigg)}\notag\\ &= \sum_{i=1}^{n} w_i \Bigg[ y_i \log\Bigg( \frac{\exp \{ {\mathbf{x}^T_i \boldsymbol{\beta}}\}}{1 + \exp \{ {\mathbf{x}^T_i \boldsymbol{\beta}\}}} \Bigg) + {(1-y_i) \log \Bigg( 1 - \frac{\exp \{ {\mathbf{x}^T_i \boldsymbol{\beta}}\}}{1 + \exp \{ {\mathbf{x}^T_i \boldsymbol{\beta}}\}} \Bigg)}\Bigg]\notag\\ &= \sum_{i=1}^{n} w_i l_i(D \mid \boldsymbol{\beta}), \label{tailored_likelihood2} \end{align*}

where $l_i(D \mid \boldsymbol{\beta})$ is the standard logistic log-likelihood function. We can further replace (2.7) into (2.9)

$$\begin{equation*}
\log(L(D \mid \boldsymbol{\beta})) = \sum_{i=1}^{n} \exp \big \{-\lambda ({\pi}_u(\mathbf{x}_i) - t)^2 \big \} l_i(D \mid \boldsymbol{\beta})
\end{equation*}$$

to see that each datapoint contributes exponentially proportional to its distance from the target threshold $t$, which summarizes the four utilities associated with binary classification problems (see 2.2). One option to proceed is by optimizing the tailored likelihood function with respect to the coefficients in an empirical risk minimization approach (Vapnik, 1998). An attractive feature of (2.9) is that this optimization is computationally efficient since we can rely on existing algorithmic tools, for example, (stochastic) gradient optimization. However, here we learn the coefficients in a Bayesian formalism.

### 2.5. Bayesian tailoring

Following Bayes Theorem, the TB posterior is

(2.10) \begin{equation*} p(\boldsymbol{\beta} \mid D) = \frac{L(D \mid \boldsymbol{\beta}) p(\boldsymbol{\beta})}{p(D)}, \label{tailored_posterior_main} \end{equation*}

where $L(D \mid \boldsymbol{\beta})$ is the tailored likelihood function given in (2.8), $p(\boldsymbol{\beta})$ is the prior on the coefficients, and $p(D) = \int L(D|\tilde{\boldsymbol{\beta}}) p(\tilde{\boldsymbol{\beta}}) d\tilde{\boldsymbol{\beta}}$, is the normalizing constant. In this work we assume a normal prior distribution for each element of $\boldsymbol{\beta}$, that is, $p(\beta_j) = \mathcal{N}(\mu_j, \sigma_j^2),$ where $\mu_j$ and $\sigma_j$ are the mean and standard deviation respectively for the $j^{\rm th}$ element of $\boldsymbol{\beta}$ ($j = 1, \dots, d + 1$). For all analysis below, we use vague priors with $\mu_j= 0$ and $\sigma_j = 100$, for all $j$.

Conveniently, we can interpret the choice of prior as a regularizer on a per-datapoint influence/importance (see Section S1). Crucially, this allows us to view the TB posterior as combining a standard likelihood function with a data-dependent prior (Section S1). Hence, even though the tailored likelihood function does not have a probabilistic interpretation the TB posterior is a proper posterior.

In the Supplementary material available at *Biostatistics* online, we provide details on the model inference and predictions steps (Section S2), the cross-validation scheme for choosing $\lambda$ (Section S3), the data-spitting strategy (Section S4), and the Markov chain Monte Carlo (MCMC) algorithm we are implementing (Section S5).

## 3. Simulations

The simulations are designed to provide insight into when TB can be advantageous compared to the standard Bayesian paradigm. Two scenarios where TB is expected to outperform standard Bayes (SB) are the absence of parallelism of the optimal decision boundaries and data contamination. A decision boundary determines distinct classification regions in the covariate space. It provides a rule to classify datapoints based on whether the datapoint’s covariate vector falls inside or outside the classification region. If a datapoint falls inside the classification region it will be labeled as belonging to class 1 (e.g., positive), if it falls outside it will be labeled as belonging to class 0 (e.g., negative). According to Bayesian decision theory the optimal decision boundaries determine the classification regions where the expected reward is maximized given prespecified misclassification costs (Duda *and others*, 2012). More specifically, we classify as positive if $\frac{\pi(\mathbf{x})}{1- \pi(\mathbf{x})} > \frac{t}{1-t}$, where $\pi(\mathbf{x})$ denotes the true class 1 probability, as in Section 2.1. Simulations 1 and 2 present two settings where the optimal decision boundaries are not parallel with their orientation changing as a function of the target threshold. Simulation 3 is an example of data contamination.

### 3.1. Simulation 1: linear decision boundaries

**Fig. 1:**
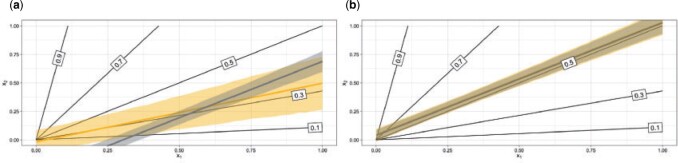
Optimal decision boundaries (black lines) for target thresholds 0.1, 0.3, 0.5, 0.7, and 0.9. Posterior mean boundaries for SB (grey) and TB (yellow) when targeting the (a) 0.3 and (b) 0.5 boundary. Shaded regions represent 90% highest predictive density (HPD) regions.

We first evaluate the performance of tailoring by extending a simulation from Hand and Vinciotti (2003). We simulate $n$ data points according to two covariates, $x_1$ and $x_2$, and assign label 1 with probability: $\theta := p(y = 1| x_1, x_2) = \frac{q x_2}{x_1 + q x_2}$ with $y \sim Bernoulli(\theta)$, $x_1, x_2 \sim \mathcal{U}(0, 1)$ and where $q$ is a scalar. The parameter $q$ determines the relative prevalence of the two classes, when $q > 1$ there are more class 1 than class 0, otherwise there are more class 0 than class 1. Figure 1 shows the optimal decision boundaries in the covariate space for a range of target thresholds using $n=5000$ and $q=1$ (which leads to a prevalence of 0.5). A key feature is that these boundaries are linear but not parallel. The absence of parallelism renders any linear model unsuitable as a global fit, but the linearity of the decision boundaries allows linear models to describe these boundaries sufficiently.

We use the decision boundaries corresponding to 0.3 and 0.5 target thresholds as exemplars. SB results in a sub-optimally estimated decision boundary for $t=0.3$ (Figure 1a). The estimated 0.3 boundary from SB is parallel to the 0.5-optimal boundary. This is expected because under this simulation setting logistic regression is bound to find a compromise model which should be linear with level lines roughly parallel to the true 0.5 boundary (where misclassification costs are equal). On the other hand, TB allows derivation of a decision boundary which is far closer to the optimum. Note the wider predictive regions of the tailoring. This is an expected consequence of our framework which we comment on in Section S9 of the Supplementary material available at *Biostatistics* online. When deriving decision boundaries under the equal costs implied by a 0.5 target threshold (Figure 1b), the two models are almost indistinguishable.

**Fig. 2:**
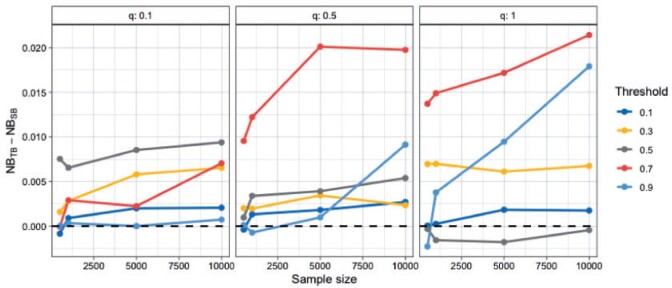
Difference in Net Benefit for samples sizes of 500, 1000, 5000, 10 000 averaged over 20 repetitions. A positive difference means TB outperforms SB. The values of 0.1, 0.5, and 1, for the $q$ parameter correspond to prevalence of around 0.15, 0.36, and 0.50, respectively.

To systematically investigate the performance of tailoring across a wide range of settings, we set-up different scenarios by varying: (i) the sample size, (ii) the prevalence of the outcome, (iii) and the target threshold. Model performance is evaluated in an independently sampled data set of size 2000. Under most scenarios tailoring outperforms standard Bayesian regression (Figure 2). The performance gains are evident even for small sample sizes. With a few exceptions (most notably $t = 0.7$ and 0.9) the advantage of tailoring is relatively stable across sample sizes. The advantage of tailoring persists even when varying the prevalence of the outcome. In fact, we see that under certain scenarios TB is superior to SB even for the 0.5 boundary. Figure S2 of the Supplementary material available at *Biostatistics* online illustrates such a scenario for $q = 0.1$, which corresponds to prevalence of 0.15. Under such class imbalance, which is common in medical applications, even when targeting the 0.5 boundary, one might want to use tailoring over standard modeling approaches.

### 3.2. Simulation 2: quadratic decision boundaries

**Fig. 3:**
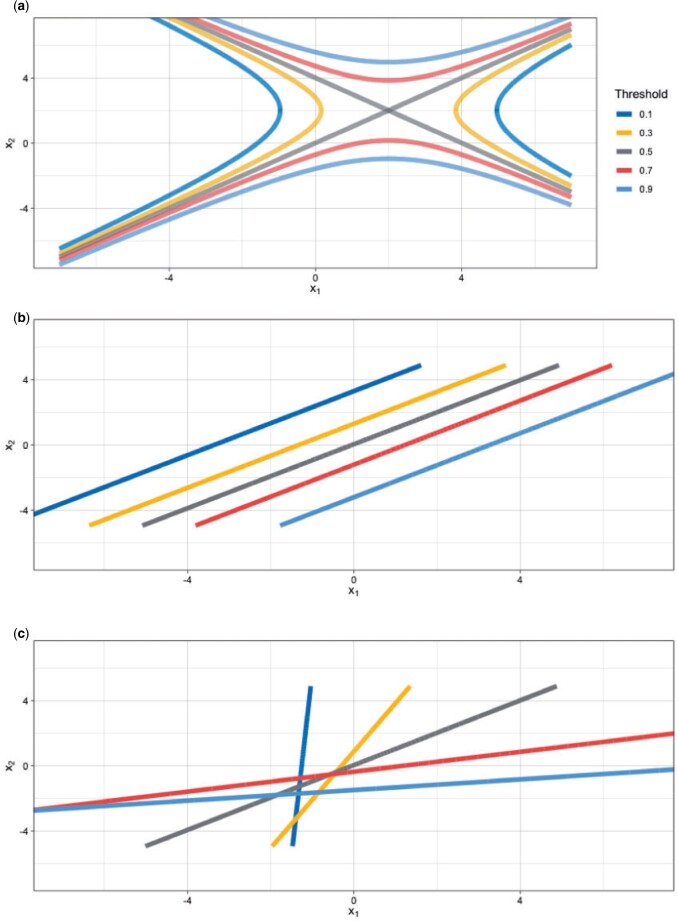
(a) Optimal decision boundaries for target thresholds 0.1, 0.3, 0.5, 0.7, 0.9. Posterior median boundaries for (b) SB, and (c) TB.

Our second simulation is a more pragmatic scenario where the optimal decision boundaries are a quadratic rather than a linear function of the covariates. The model is of the form

$$\begin{eqnarray*}
\mathbf{x} | y = 1
& \sim & \mathcal{N} \left(\left[\begin{array}{c}
1\\
0\\
\end{array}\right], \left[ \begin{array}{cc}
1, 0 \\
0, 2
\end{array}\right] \right)\\
\mathbf{x} | y = 0
& \sim & \mathcal{N} \left(\left[\begin{array}{c}
0\\
1\\
\end{array}\right],\left[ \begin{array}{cc}
2, 0 \\
0, 1
\end{array} \right] \right),
\end{eqnarray*}$$

where $\mathbf{x} = (x_1, x_2)^T$ contains the two continuous-valued predictors. The marginal probabilities of the outcome are equal, that is, $p(y = 0) = p(y = 1) = 0.5$. In this case of unequal covariance matrices, the optimal decision boundaries are a quadratic function of $\mathbf{x}$ (Figure 3a) (Duda *and others* (2012), Chapter 2). A linear model, like the one we implement is suboptimal. Nevertheless, this example allows us to demonstrate in an analytically tractable way the advantage of tailoring and it allows us to explore a broader array of generic simulation examples, since arbitrary Gaussian distributions lead to decision boundaries that are general hyperquadrics.

Figure 3(b) and (c) shows the posterior median decision boundaries for SB and TB using $n = 5000$ under the data generating model described above, and for a range of target thresholds. It is clear that the direction of the optimal decision boundary is a function of the costs. The parallel decision boundaries obtained by applying different thresholds to the standard logistic predictions are clearly not an optimal solution when comparing against the optimal boundaries depicted in Figure 3(a). Although limited to estimation of linear boundaries, tailoring is able to adapt the angle of the boundary to better approximate the optimal curves. One exception in comparative performance is the 0.5 threshold which is estimated perfectly for both models. This is expected, since the standard logistic model targets the 0.5 boundary.

**Fig. 4:**
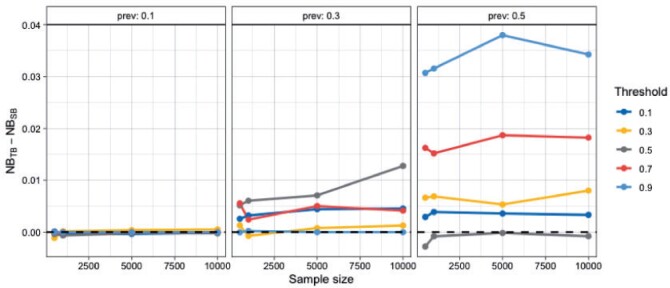
Difference in Net Benefit for samples sizes of 500, 1000, 5000, 10 000 averaged over 20 repetitions. A positive difference means TB outperforms SB. Each grid corresponds to a different prevalence setting.

As before, we investigate the performance of tailoring across a wide range of settings, by varying: (i) the sample size, (ii) the prevalence of the outcome, and (iii) the target threshold. Performance is evaluated in an independently sampled test set of size 2000. Figure 4 shows the difference in NB between TB and SB. Tailoring performs similarly or better than standard regression across all target thresholds for prevalence scenarios 0.3 and 0.5. For 0.1 the two models are closely matched. A further comparison with a non-linear model, namely Bayesian Additive Regression Trees (BART) (Sparapani *and others*, 2021) is detailed in the supplementary material (Section S7). Briefly, TB demonstrated equivalent or better performance than BART at the clinically relevant lower disease prevalences of 0.1 and 0.3, indicating that the benefits offered by TB cannot be matched simply by switching to a non-linear modeling framework.

### 3.3. Simulation 3: Data contamination

**Fig. 5:**
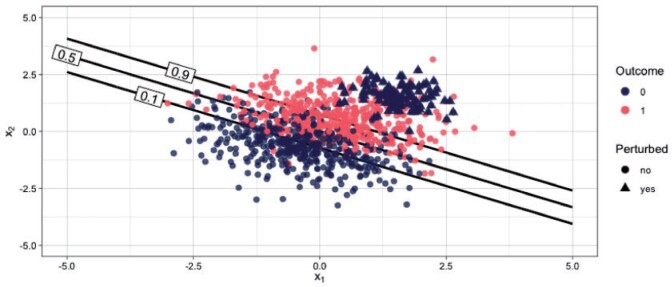
Single realization from contaminated distribution with $10\%$ corrupted datapoints. Data ($n = 1000$) with labels 0 and 1 are shown in blue and red, respectively. The corrupted data points are depicted with triangles on the upper right-hand corner of the data cloud. The lines corresponds to target thresholds 0.1, 0.5, and 0.9.

Our third simulation scenario demonstrates the robustness of tailoring to data contamination, that is, the situation in which a fraction of the data have been mislabeled. The data generating model is a logistic regression with a large fraction of mislabeled datapoints. We simulate $d = 2$ covariates and $n = 1000$ datapoints. Figure 5 depicts a scenario with $10\%$ of datapoints mislabeled among those with high values of both covariates, that is, among the upper right hand side of the data cloud. For each covariate, $1000$ values are independently drawn from a standard Gaussian distribution. Denoting the coefficient vector by $\boldsymbol{\beta} \in \mathbb{R}^{3}$ with values $\boldsymbol{\beta} = (0, 2, 3)$ (the first value corresponds to the intercept term) we simulate the outcome vector as $y \sim Bernoulli \Big( \frac{\exp \{ {\mathbf{x}^T \boldsymbol{\beta}} \}}{1 + \exp\{ {\mathbf{x}^T \boldsymbol{\beta}}\}} \Big)$, where $\mathbf{x} = (1, x_1, x_2)^T$. We then corrupt the data with class 0 datapoints, that is, we set $y := 0$ for $\psi n$ datapoints where $\psi$ is the fraction of contamination taking values $5\%, 10\%, 15\%, 20\%,$ and $30\%$. The covariates are generated from equivalent and independent normal distributions, specifically $x_1, x_2 \sim \mathcal{N}(1.5, 0.5)$. This type of contamination framework has been popularized by Huber (1964, 1965) and used extensively to study the robustness of learning algorithms to adversarial attacks in general (Balakrishnan *and others*, 2017; Diakonikolas *and others*, 2019; Prasad *and others*, 2018; Osama *and others*, 2020) and medical applications (Paschali *and others*, 2018).

**Fig. 6:**
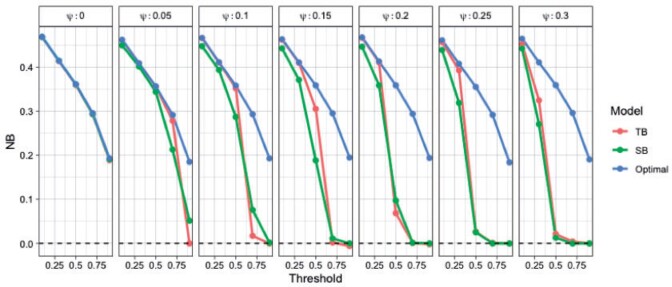
Net Benefit of tailoring (red) and standard regression (green) compared to optimal classification (blue) averaged over 20 repetitions. Each grid corresponds to different contamination fraction.

We derive the optimal NB based on the true probability score in an independent non-contaminated test data set of size $n = 2000$. Figure 6 shows the results for various contamination fractions. For most fractions TB outperforms SB. As the contamination fraction gets larger the performance of both models degrades, but standard regression degrades at a faster rate. Tailoring can accommodate various degrees of contamination better than standard regression, while generally never resulting in poorer performance.

Note that under no contamination (i.e., $\psi = 0$, first panel Figure 6) SB is an optimal classifier, since the optimal decision boundaries are parallel straight lines (Figure 5). However, for all other scenarios even a data corruption as small as 5% results in poor performance under SB for target thresholds $>0.5$. On the contrary, tailoring maintains stable performance and close to the optimal for $t< 0.5$, for up to 15% of mislabeled datapoints.

## 4. Real data applications

We evaluate the performance of TB on three real-data applications involving a breast cancer prognostication task (Section 4.1), a cardiac surgery prognostication task (Section 4.2) and a breast cancer tumor classification task (Section S8 of the Supplementary material available at *Biostatistics* online). Overall, our empirical results demonstrate the improvement in predictive performance when taking into consideration misclassification costs during model training.

### 4.1. Real data application 1: Breast cancer prognostication

Here, we apply the TB methodology to predict mortality after diagnosis with invasive breast cancer. The training data is based on 4718 estrogen receptor positive subjects diagnosed in East Anglia, UK between 1999 and 2003. The outcome modeled is 10-year mortality. The covariates are age at diagnosis, tumor grade, tumor size, number of positive lymph nodes, presentation (screening vs. clinical), and type of adjuvant therapy (chemotherapy, endocrine therapy, or both). We use 20% of the data as design and the rest as development set (see Figure S1 of the Supplementary material available at *Biostatistics* online), repeating the design/development set split $m = 5$ times. The entire train data is used to fit SB. Both models are evaluated in an independent test set consisting of 3810 subjects. Detailed information on the data sets can be found in Karapanagiotis *and others* (2018).

An important part of the TB methodology is the choice of $t$. In breast cancer, accurate predictions are decisive because they guide treatment. In clinical practice, treatment is given if it is expected to reduce the predicted risk by at least some pre-specified magnitude. For instance, clinicians in the Cambridge Breast Unit (Addenbrooke’s Hospital, Cambridge, UK) currently use the absolute 10-year survival benefit from chemotherapy to guide decision making for adjuvant chemotherapy as follows: $<$3% no chemotherapy; 3%–5% chemotherapy discussed as a possible option; $>$5% chemotherapy recommended (Down *and others*, 2014). Following previous work (Karapanagiotis *and others*, 2018), we assume that chemotherapy reduces the 10-year risk of death by 22% (Peto *and others*, 2012). Then, a risk reduction between 3% and 5%, corresponds to target thresholds between 14% and 23%. Hence, we explore misclassification cost ratios corresponding to $t$ in the range between 0.1 and 0.5.

**Fig. 7:**
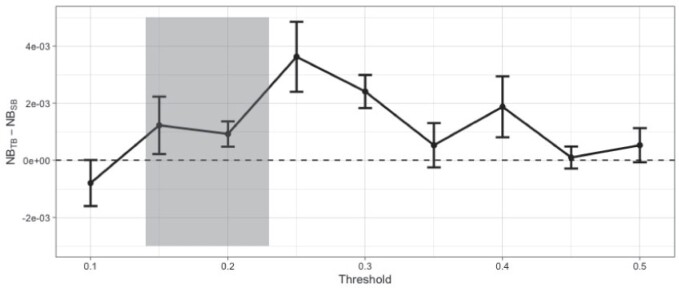
Difference in Net Benefit for various $t$ values evaluated on the test set. Error bars correspond to one standard error of the difference. That is, denoting the difference in Net Benefit $D^i = NB^i_{TB} - NB^i_{SB}$ with $i = 1, \dots, m = 5$ for each $t$ then the standard error of the difference is $SE_D = \sqrt{\frac{\sum (D^i - \bar{D})^2}{m(m-1)}}$, where $\bar{D} = \sum_i D^i / m$. This accounts for the fact that both models have been evaluated on the same data. The units on the y axis may be interpreted as the difference in benefit associated with one patient who would die without treatment and who receives therapy. The 0.14–0.23 shaded area on the x axis corresponds to 3%–5% absolute risk of death reduction with and without chemotherapy. These are the risk ranges where chemotherapy is discussed as a treatment option.

Figure 7 shows the difference in NB between the two models averaged over the five splits. We see TB outperforms SB for most target thresholds, especially where decisions about adjuvant chemotherapy are made. Compared to SB, tailoring achieves up to 3.6 more true positives per 1000 patients (when $t = 0.15$), which is equivalent to having 3.6 more true positives per 1000 patients for the same number of unnecessary treatments.

**Fig. 8:**
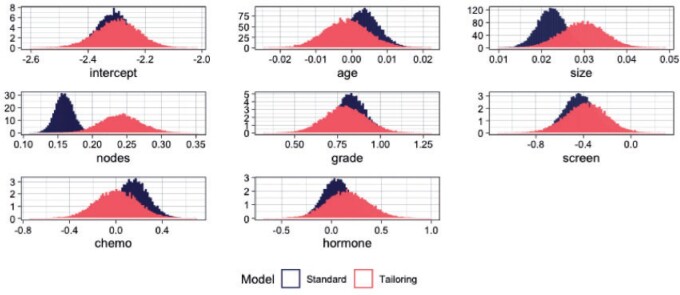
Marginal density plots of posterior parameters for $t = 0.15$ for SB (blue) and TB (red).

Next, we examine the effect of tailoring on the posterior distributions of the coefficients. As an exemplar, we use the posterior samples for the model corresponding to $t = 0.15$ (Figure 8). We see that tailoring affects both the location and spread of the estimates compared to standard modeling. First, note the wider spread of tailoring compared to the standard models. Second, the tailored posteriors are centered on different values. The most extreme example is the coefficient for the number of nodes. Under tailoring it has a stronger positive association with the risk of death. To quantify the discrepancy between the posteriors of the two models Table 1 shows estimates of the overlapping area between the posteriors for each covariate. These range from 3% to 70%. The relative shifts in magnitude of the effect sizes indicates different relative importance of the covariates in terms of their contribution to the predictions from the two models.

**Table 1.** Overlapping area of posterior distributions for each coefficient based Gaussian kernel density estimations (Pastore and Calcagnì, 2020).

Covariate	Posterior overlap (%)
Nodes	3.05
Size	23.46
Chemo	41.92
Age	48.78
Hormone	57.76
Grade	62.66
Screen	69.94

### 4.2. Real data application 2: Cardiac surgery prognostication

For our second case study, we investigate whether TB allows for better predictions, and consequently improved clinical decisions for patients undergoing aortic valve replacement (AVR). Cardiac patients with severe symptomatic aortic stenosis are considered for surgical AVR (SAVR). Given that SAVR is typically a high-risk procedure, transcatheter aortic valve implantation (TAVI) is recommended as a lower risk alternative but it is associated with higher rates of complications (Baumgartner *and others*, 2017). The European System for Cardiac Operative Risk Evaluation (EuroSCORE) is routinely used as a criterion to choose between SAVR and TAVI (Roques *and others*, 2003). EuroSCORE is an operative mortality risk prediction model which takes into account 17 covariates encompassing patient-related, cardiac and operation-related characteristics. It was first introduced by Nashef *and others* (1999) and it has been updated in 2003 (Roques *and others*, 2003) and 2012 (Nashef *and others*, 2012). Published guidelines recommend TAVI over SAVR if a patient’s predicted mortality risk is above 10% (Baumgartner *and others*, 2017) or 20% (Vahanian *and others*, 2008). Here, we compare the performance of TB with EuroSCORE and SB given these target thresholds.

**Fig. 9:**
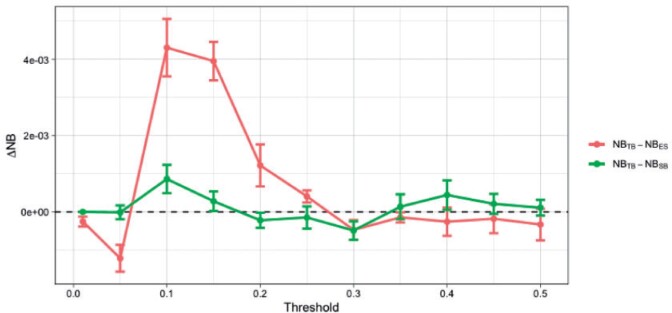
Difference in Net Benefit ($\Delta$NB) between TB and EuroSCORE (ES) (red), and between TB and SB (green) for various target thresholds evaluated on the test set. Error bars correspond to one standard error of the difference (see caption of Figure 7 for details).

We use data ($n$ = 9031) from the National Adult Cardiac Surgery Audit (UK) collected between 2011 and 2018. We use 80% of the data for training and the rest for testing, repeating the train/test set split $m = 5$ times. For this data a design set to estimate $\pi_u(\mathbf{x}_i)$ is not necessary (see Figure S1 of the Supplementary material available at *Biostatistics* online) but instead we use the predictions from EuroSCORE (Roques *and others*, 2003). We add an extra step of re-calibration to account for the population/time drift (Cox, 1958; Miller *and others*, 1993). Figure 9 presents the results. We see TB outperforms both EuroSCORE and SB when targeting the 0.1 threshold, and only EuroSCORE at $t = 0.2$.

**Fig. 10:**
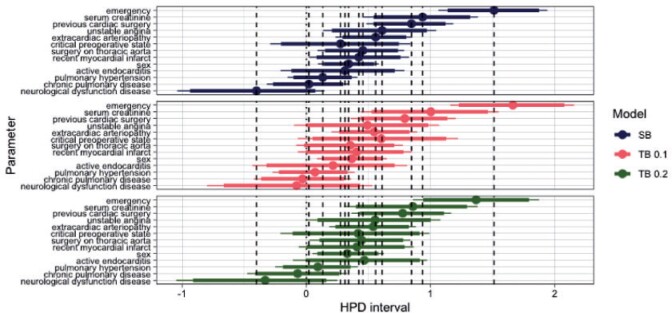
Highest posterior density (HPD) regions for the parameters. Dots represent medians, and thick and thin lines represent 90 and the 95% of the HPD regions, respectively. The dashed vertical lines pass through the posterior median values of the SB parameters.

We further investigate the effect of tailoring to individual parameters. Figure 10 shows the highest posterior density (HPD) regions for a subset of the covariates under SB and TB for $t= 0.1$ and $0.2$. As in the previous case study, under tailoring the regions are generally wider and are centered on different values. For instance, compared to SB under both $t = 0.1$ and 0.2 the posteriors of critical operative state and unstable angina are shifted towards the same direction (positive for critical operative state and negative for unstable angina). Contrast these with the posterior of emergency that compared to SB it is centered on more positive values under $t =0.1$ and more negative under $t =0.2$. On the contrary, extracardiac arteriopathy, recent myocardial infarct, and sex are centered on similar values across the three models. This once more exemplifies the change in the contribution of some covariates towards the predicted risks when taking into account misclassification costs.

## 5. Discussion

In this work, we present Tailored Bayes, a framework to incorporate misclassification costs into Bayesian modeling. We demonstrate that our framework improves predictive performance compared to standard Bayesian modeling over a wide range of scenarios in which the costs of different classification errors are unbalanced.

The methodology relies solely on the construction of the datapoint-specific weights (see (2.7)). In particular, we need to specify $t$, the grid of $\lambda$ values for the CV, a model to estimate $\pi_u(\mathbf{x}_i)$ and the weighting function, $h$. For some applications there may be a recommended target threshold, $t$. For instance, UK national guidelines recommend that clinicians use a risk prediction model (QRISK2; Hippisley-Cox *and others*, 2008) to determine whether to prescribe statins for primary prevention of cardiovascular disease (CVD) if a person’s CVD risk is 10% or more (NICE, 2016). When guidelines are not available, the specification of $t$ is inevitably subjective, since it reflects the decision maker’s preferences regarding the relative costs of different classification errors. In practice, eliciting these preferences may be challenging, despite the numerous techniques that have been proposed in the literature to help with this (e.g., Tsalatsanis *and others*, 2010; Hunink *and others*, 2014). In such situations, we advocate fitting the model for a range of plausible $t$ values that reflect general decision preferences. For example, research in both mammographic (Schwartz *and others*, 2000) and colorectal cancer screening (Boone *and others*, 2013) has shown that healthcare professionals and patients alike greatly value gains in sensitivity over loss of specificity. For additional examples on setting $t,$ see Vickers *and others* (2016) and Wynants *and others* (2019). Further examples in which benefits and costs associated with an intervention (as well as with patients’ preferences) are taken into account, are provided by Manchanda *and others* (2016), Le and others (2017), Watson *and others* (2020).

We discuss the remaining elements for the construction of the weights in Section S9 of the Supplementary material available at *Biostatistics* online. There we define the effective sample size for tailoring, $ESS_T$, and showcase how to use it to set the upper limit for the grid of $\lambda$ values. In addition, we show our framework is robust to miscalibration of $\pi_u(\mathbf{x}_i)$ and the choice of $h$. The framework is therefore flexible, allowing many ways for the user to specify the weights.

In contrast to the work of Hand and Vinciotti (2003), our approach is framed within the Bayesian formalism. Consequently, the tailored posterior integrates the attractive features of Bayesian inference—such as flexible hierarchical modeling, the use of prior information and quantification of uncertainty—while also allowing for tailored inference. Quantification of uncertainty is critically important, especially in healthcare applications (Begoli *and others*, 2019; Kompa *and others*, 2021). Whilst two (or more) models can perform similarly in terms of aggregate metrics (e.g., area under ROC curve) they can provide very different individual (risk) predictions for the same patient (Pate *and others*, 2019; Li *and others*, 2020). This can ultimately lead to different decisions for the individual, with potential detrimental effects. Uncertainty quantification can mitigate this issue since it allows the clinician to abstain from utilizing the model’s predictions. If there is high predictive uncertainty for an individual, the clinician can discount or even disregard the prediction.

**Fig. 11:**
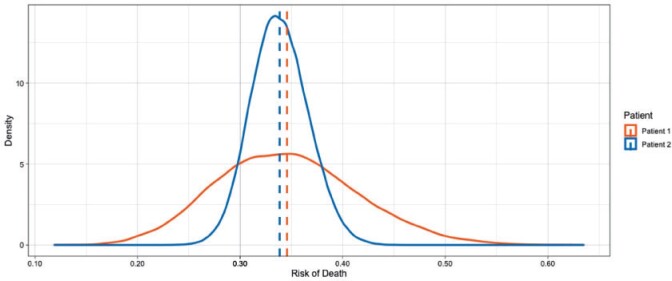
Predictive uncertainty for the risk of death in two patients. These posterior predictive distributions reflect the range of risks assigned to these patients, and the mean risk is shown as vertical lines. Despite the fact that both patients have similar mean risks, we may be more inclined to trust the predictions for patient 2 given the lower amount of uncertainty associated with that prediction.

To illustrate this point, we use the SB posterior from the breast cancer prognostication case study. The posterior predictive distributions for two patients are displayed in Figure 11. The average posterior risk for each patient is indicated by the vertical line at 34 and 35%, respectively. Based solely on these average estimates chemotherapy should be recommended as a treatment option to both patients (see Section 4.1). It is clear, however, that the predictive uncertainty for these two patients is quite different, as the distribution of risk for patient 1 is much more dispersed than the distribution for patient 2. One way to quantify the predictive uncertainty would be to calculate the standard deviation of these distributions, which are 6.9% and 2.8% for patient 1 and patient 2, respectively. Even though both estimates are centered at similar values the predictive uncertainty for patient 1 is more than two times higher than patient 2. Using this information, we could flag patient 1 as needing more information before making a clinical decision.

A few related comments are in order. In this work, we use vague Gaussian priors, but they could be replaced with other application-specific distribution choices. For instance, in the case of high-dimensional data another option could be the sparsity-inducing prior used by Bayesian lasso regression (Park and Casella, 2008). Furthermore, we can easily incorporate external information in a flexible manner, through $\pi_u(\mathbf{x})$, in addition to the prior on the coefficients. If a well-established model exists, then it is natural to consider using it to improve the performance of an expanded model. We have implemented such an approach in Section 4.2. Cheng *and others* (2019) propose several approaches for incorporating published summary associations as prior information when building risk models. A limitation of their approaches is the requirement for a parametric model, that is, information on regression coefficients. Our method does not have any restriction on the form of $\pi_u(\mathbf{x})$, it can arise from a parametric or non-parametric model.

We note that we opted to use the same set of covariates, $\mathbf{x}$, to estimate both $\pi_{w_i}(\mathbf{x};\boldsymbol{\beta})$ and $\pi_u(\mathbf{x})$. This does not need to be the case. If available, we could instead use another set of covariates, say $\mathbf{Z}$ to estimate $\pi_u(\mathbf{z})$. The set $\mathbf{Z}$ could be a superset or a subset of $\mathbf{X}$ or the two sets could be completely disjoint. We also note that in this work we focus on linear logistic regression to showcase the methodology (linear refers to linear combinations of the covariates). This is because it is widely utilized and allows analytical and computational tractability. Nevertheless, we would stress that our framework is generic, and not restricted to linear logistic regression. It can accommodate a wide range of modeling frameworks, from linear to non-linear and from classical statistical approaches to state-of-the-art machine learning algorithms. As a result, future work could consider such extensions to non-linear models. Also, future work could consider the advantages of a joint estimation, that is, both steps, stage 1 (estimation of weights) and stage 2 (estimation of weighted prediction probabilities) jointly. A further direction is the extension of the framework to high-dimensional settings.

To conclude, in response to recent calls for building clinically useful models (Chatterjee *and others*, 2016; Shah *and others*, 2019), we present an overarching Bayesian learning framework for binary classification where we take into account the different benefits/costs associated with correct and incorrect classifications. The framework requires the modelers to first think of how the model will be used and the consequences of decisions arising from its use—which we would argue should be a prerequisite for any modeling task. Instead of fitting a global, agnostic model and then deploying the result in a clinical setting we propose a Bayesian framework to build towards models tailored to the clinical application under consideration.

## 6. Software

The R code used for the experiments in this article has been made available as an R package, TailoredBayes, on Github: https://github.com/solonkarapa/TailoredBayes.

## Supplementary Material

kxab023_Supplementary_Data

## Footnotes

^1^ Note this example constitutes a simplification of the problem aimed to introduce the main idea of the article, that is, in some applications the false positives and negatives have different costs. Hence, we are not considering the negative effects of toxicity of chemotherapy, overdiagnosis/unnecessary treatment for certain cancers, quality of life issues, etc.

^2^ The statement is equivalent to the following: Assume that not treating an individual with the outcome (false negative) is 9 times worse than treating unnecessarily a healthy individual (false positive). Both statements result in the same harm-to-benefit ratio.
