# Supplementary material for: Tailored Bayes: a risk modeling framework under unequal misclassification costs
Source: Biostatistics. Author manuscript; Available in PMC 2022 Dec 15. (PMC9748575; doi:10.1093/biostatistics/kxab023)
Supplement: Supplementary File [file EMS140632-supplement-Supplementary_File.pdf]

Supplementary Materials for "Tailored Bayes: a risk modeling framework under unequal misclassification costs"

Solon Karapanagiotis, Umberto Benedetto, Sach Mukherjee, Paul D. W. Kirk, Paul J. Newcombe

S1. Interpretation of the TB prior (and posterior)

Here we show the prior in TB can be interpreted as a regularizer on a per-datapoint influence/importance. First, we slightly modify notation and consider data $D_i = (X_i, Y_i)$ as a copy of a random variable $D = (X, Y) \in \mathbb{R}^d \times \{0, 1\}$. Then, let $\mathcal{L}(D_i|\boldsymbol{\beta})$ be the standard likelihood contribution of datapoint i $(i = 1, \ldots, n)$. The TB posterior, up to a normalising constant, is

$$p(\boldsymbol{\beta}|D) \propto \prod_{i=1}^{n} \mathcal{L}(D_i|\boldsymbol{\beta})^{w_i} p(\boldsymbol{\beta}). \tag{S1}$$

Following Walker and Hjort (2001) we can view (S1) as combining the original likelihood function with a *data-dependent* prior that is divided by a portion of the likelihood. To see this, we first define the data-dependent prior as

$$\frac{p(\boldsymbol{\beta})}{\prod_{i=1}^{n} \mathcal{L}(D_i|\boldsymbol{\beta})^{1-w_i}}$$

which corresponds to

$$p(\boldsymbol{\beta}|D) \propto \prod_{i=1}^{n} \mathcal{L}(D_i|\boldsymbol{\beta}) \frac{p(\boldsymbol{\beta})}{\prod_{i=1}^{n} \mathcal{L}(D_i|\boldsymbol{\beta})^{1-w_i}} = \prod_{i=1}^{n} \mathcal{L}(D_i|\boldsymbol{\beta})^{w_i} p(\boldsymbol{\beta}),$$

which is seen to coincide with (S1). This data-dependent downweighting of the prior reduces the weights of those parameter values that "track the data too closely" (Linero and Yang, 2018).

S2. Model inference and prediction

To sample from the TB posterior we use Markov Chain Monte Carlo (MCMC) (see Section S5 for details on the computational scheme). We obtain S posterior samples $\{\boldsymbol{\beta}^s\}_{s=1}^{S}$, where $\boldsymbol{\beta}^s = (\beta_1^s, \ldots, \beta_{d+1}^s)$. We use the posterior samples to approximate the predictive density for test data $\mathbf{x}_*$

$$\begin{aligned} p(\pi(\mathbf{x}_*) \mid \mathbf{x}_*, D) &= \int p(\pi(\mathbf{x}_*) \mid \mathbf{x}_*, \boldsymbol{\beta}) p(\boldsymbol{\beta} \mid D) d\boldsymbol{\beta} \\ &\approx \frac{1}{S} \sum_{s=1}^{S} p(\pi(\mathbf{x}_*) \mid \mathbf{x}_*, \boldsymbol{\beta}^s). \end{aligned} \tag{S2}$$

To calculate point predictions we summarise (S2) by using the posterior predictive mean,

$$\hat{\pi}(\mathbf{x}_*) = \int \pi(\mathbf{x}_*) p(\pi(\mathbf{x}_*) \mid \mathbf{x}_*, D) d\pi(\mathbf{x}_*), \tag{S3}$$

which is used as a plug-in into (2.5) in the main manuscript. We also use Bayesian inference for the estimation of $\pi_u(\mathbf{x})$ so $\pi(\mathbf{x}_*)$ can be conceptually replaced by $\pi_u(\mathbf{x})$ in eq (S2) and (S3) with the caveat that they are estimated in different subsets of the data (see Section S4 for the data splitting stategy we are implementing).

S3. Cross-validation to choose λ

We use stratified K-fold cross-validation (CV) to choose λ in (2.7) in the main manuscript. The stratification ensures the prevalence of the outcome is the same in each fold. In K-fold CV, the data is partitioned into K subsets $D_{(k)}$, for $k = 1, \dots, K$ and then the model is fit separately to each training set $D_{(-k)}$ thus yielding a posterior distribution $p(\boldsymbol{\beta} \mid D_{(-k)})$. When calculating the predictive performance of the model the data of the kth fold is used as test data. The predictive density for $\mathbf{x}_*$, if it is in subset k, is

$$\begin{aligned} p(\pi(\mathbf{x}_*) \mid \mathbf{x}_*, D_{(-k)}) &= \int p(\pi(\mathbf{x}_*) \mid \mathbf{x}_*, \boldsymbol{\beta}) p(\boldsymbol{\beta} \mid D_{(-k)}) d\boldsymbol{\beta} \\ &\approx \frac{1}{S} \sum_{s=1}^{S} p(\pi(\mathbf{x}_*) \mid \mathbf{x}_*, \boldsymbol{\beta}^s). \end{aligned} \tag{S4}$$

and the posterior predictive expectation is $\hat{\pi}(\mathbf{x}_*) = \int \pi(\mathbf{x}_*) p(\pi(\mathbf{x}_*) \mid \mathbf{x}_*, D_{(-k)}) d\pi(\mathbf{x}_*)$ which is used as a plug-in into (2.5) in the main manuscript to calculate the K-fold CV estimate of NB in the kth fold, $\text{NB}_{(k)}$. We choose λ as

$$\lambda^* = \arg\max_{\lambda} \frac{1}{K} \sum_{k=1}^{K} \text{NB}_{(k)}.$$

We use $K = 5$ for the all analysis. In practice, we have seen the results are insensitive to the choice of K.

S4. Data splitting strategy

To avoid overfitting due to the estimation of both $\pi_u(\mathbf{x}_i)$ and $\pi_{w_i}(\mathbf{x}_i; \boldsymbol{\beta})$ from the same dataset we use the following data splitting process (Figure S1 and Pseudo-Code 1). First, the data is split into training and testing sets. This step is avoided if we already have an independent test set (Section 4.1 main manuscript) or if data is simulated (Section 3 main manuscript). The train set is subsequently split again into design (20%) and development (80%). The design part is used to estimate $\pi_u(\mathbf{x}_i)$. The development part is used to choose λ (with 5-fold stratified CV, see Section S3 for details). After choosing a λ value the model is fit to the entire development part, and it is with respect to the posterior from this final fit credible intervals are generated in the analyses. Finally, the test set is used to validate the performance of the model. We opted for this three-way splitting strategy, design-development-test set, because we have large datasets available but any other method such as leave-one-out, (nested) cross-validation, or bootstrap methods could be used.

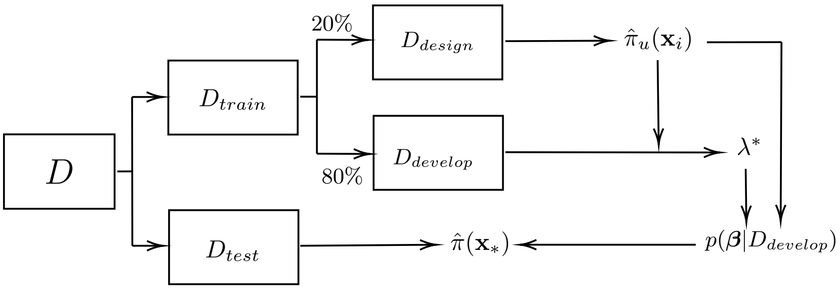

Fig. S1: The data splitting strategy. The dataset, D, is split into train (D_{train}) and test (D_{test}) sets. The train set is subsequently split again into design (D_{design}) (20%) and development ($D_{develop}$) (80%). The design part is used to estimate $\hat{\pi}_u(\mathbf{x}_i)$. The development part is used to choose λ^* (5-fold CV, see Section S3 for details). After choosing a λ^* value the model is fit to the entire development part, obtaining the posterior, $p(\boldsymbol{\beta}|D_{develop})$. Finally, the test set is used to create predictions, $\hat{\pi}(\mathbf{x}_*)$ (this is the posterior predictive mean defined in Section S2.)

Pseudo-code 1: The TB algorithm.

input: D: dataset,
K: number of folds for the cross-validation,
λ: tuning parameter,
Model$_u$: model to estimate $\pi_u(\mathbf{x}_i)$,
Model$_w$: model to estimate $\pi_{w_i}(\mathbf{x}_i;\boldsymbol{\beta})$
procedure TB(D, K, λ, Model$_u$, Model$_w$)

1. D_{design}, $D_{develop}$, $D_{test} \leftarrow D$ ▷ randomly split D into design, development and test sets
2. $f_u \leftarrow \text{Model}_u(D_{design})$ ▷ train a model on D_{design} to estimate a function f_u [a]
3. $\hat{\pi}_u(\mathbf{x}_i) \leftarrow f_u(\mathbf{x}_i)$ ▷ apply the function f_u to $\mathbf{x}_i$ where $i \in D_{develop}$ to obtain $\hat{\pi}_u(\mathbf{x}_i)$ [b]
4. $\lambda^* \leftarrow \text{CV}(D_{develop}, K, \lambda)$ ▷ use cross-validation to obtain λ^* [c]
5. $f \leftarrow \text{Model}_w(D_{develop}, \lambda^*)$ ▷ train a model on $D_{develop}$ to estimate a function f [d]
6. $\hat{\pi}(\mathbf{x}_*) \leftarrow f(\mathbf{x}_*)$ ▷ apply the function f to $\mathbf{x}_* \in D_{test}$ to obtain $\hat{\pi}(\mathbf{x}_*)$[e]
7. **end procedure**

output: $\hat{\pi}(\mathbf{x}_*)$ ▷ the posterior predictive mean. Additionally, the posterior distribution of f can be returned

[a] In this work, Model$_u$ is the standard Bayesian logistic regression and $f_u(\mathbf{x}) := \mathbf{x}^T\boldsymbol{\beta}_u$. Hence, we are estimating the posterior of $\boldsymbol{\beta}_u$.
[b] $\hat{\pi}_u(\mathbf{x}_i)$ is the posterior predictive mean, which integrates over the uncertainty in $\boldsymbol{\beta}_u$, see section S2 for formal definition.
[c] The CV function performs K-fold cross-validation using the development set as input. For the pre-specified λ values returns λ^*, the value that gives the highest average NB, see section S3 for formal definition.
[d] In this work, Model$_w$ is the tailored Bayesian logistic regression (Sections 2.3 and 2.4, main manuscript) and $f(\mathbf{x}) := \mathbf{x}^T\boldsymbol{\beta}$.
[e] $\hat{\pi}(\mathbf{x}_*)$ is the posterior predictive mean, which integrates over the uncertainty in $\boldsymbol{\beta}$, see section S2 for formal definition.

S5. Computational scheme

For all analysis in this report we use MCMC which has become a very important computational tool in Bayesian statistics since it allows for Monte Carlo approximation of complex posterior distributions where analytical or numerical integration techniques are not applicable. The Markov chain is constructed using random walk Metropolis-Hastings updates (Brooks *and others*, 2011). We give a brief overview of the algorithm.

The target distribution is $p(\boldsymbol{\beta}|D)$ (see (2.10) main manuscript). The sampling scheme starts at an initial set of parameter values, denote these $\boldsymbol{\beta}^0$. To sample the next set of parameters, which we denote $\boldsymbol{\beta}^1$, we propose moving from the current state to another set of parameter values, $\boldsymbol{\beta}^{new}$, by using a proposal function $q(\boldsymbol{\beta}^{new}|\boldsymbol{\beta})$. We then accept these proposed values as the next sample with probability equal to the Metropolis-Hastings ratio:

$$\text{MHR}(\boldsymbol{\beta},\boldsymbol{\beta}^{new}) = \frac{L(D|\boldsymbol{\beta}^{new})p(\boldsymbol{\beta}^{new})}{L(D|\boldsymbol{\beta})p(\boldsymbol{\beta})} \times \frac{q(\boldsymbol{\beta}|\boldsymbol{\beta}^{new})}{q(\boldsymbol{\beta}^{new}|\boldsymbol{\beta})}, \tag{S5}$$

where $L(D|\boldsymbol{\beta})$ is the tailored likelihood and $p(\boldsymbol{\beta})$ the prior, given in Sections 2.4 and 2.5 of the main manuscript. The proposed move is accepted with probability

$$\alpha(\boldsymbol{\beta},\boldsymbol{\beta}^{new}) = min(1, \text{MHR}(\boldsymbol{\beta},\boldsymbol{\beta}^{new})).$$

If this new set of values is accepted, the proposed set is accepted as $\boldsymbol{\beta}^1$; otherwise, the sample value remains equal to the current sample value, i.e., $\boldsymbol{\beta}^1 = \boldsymbol{\beta}^0$. The proposal function is Gaussian, i.e., $q \sim \mathcal{N}(\boldsymbol{\beta}, \boldsymbol{I}sd)$ where sd is chosen to yield an acceptance rate ≈ 0.24 (Brooks *and others*, 2011). In the current version of the algorithm all parameters are updated jointly.

S6. Supplementary Figures

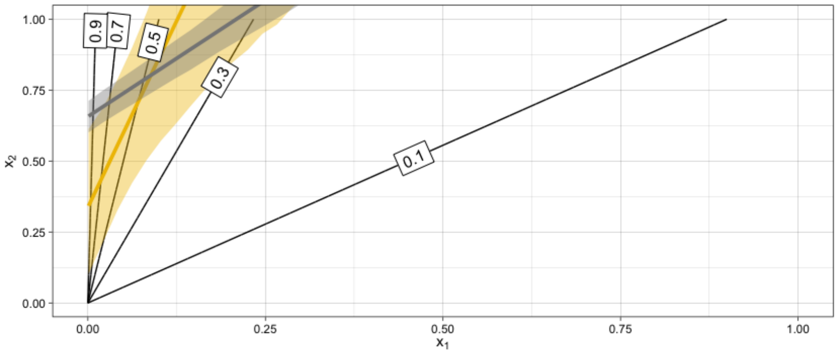

Fig. S2: Single realisation from model in Section 3.1 with $q = 0.1$ corresponding to prevalence of around 0.15. Optimal decision boundaries (black lines) for target thresholds 0.1, 0.3, 0.5, 0.7, 0.9. Posterior mean boundaries for SB (grey) and TB (yellow) when targeting the 0.5 boundary. Shaded regions represent 90% highest predictive density (HPD) regions.

S7. Comparison with BART

Given the non-linear decision boundaries of the simulation scenario in Section 3.2, we further compare TB with a standard non-linear Bayesian model. We use logistic Bayesian Additive Regression Trees (BART) as implemented in the BART package version 2.9 (lbart() function) (Sparapani *and others*, 2021).

Figure S3 shows the difference in NB between TB and BART. Under the 0.5 prevalence scenario BART performs better than TB except at $t = 0.9$. On the other hand, TB performs better or no worse than BART under prevalence scenarios 0.1 and 0.3. This is noteworthy as these prevalence scenarios are common in medical applications. Together with the results from Figure 4 (main manuscript), we conclude that for this simulation scenario, TB, albeit implemented as a linear model, mitigates some of the advantages of a non-linear one, such as BART. Note that an additional comparison of interest would be BART with a tailored BART implementation. We leave this for future work.

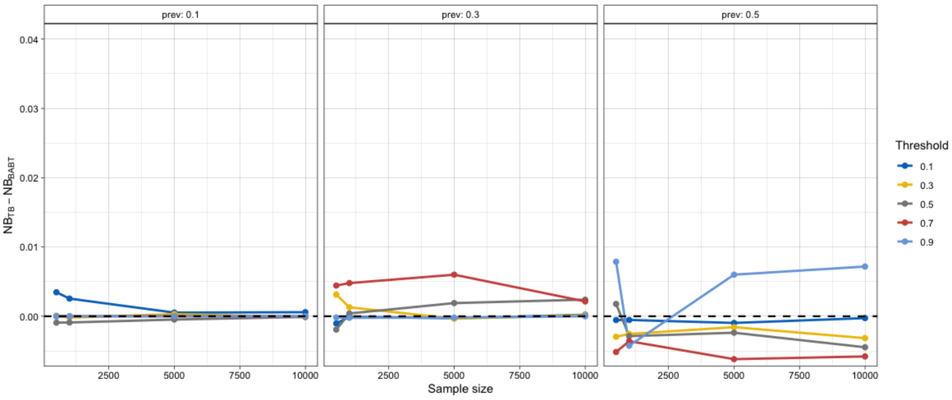

Fig. S3: Difference in Net Benefit for samples sizes of 500, 1000, 5000, 10000 averaged over 20 repetitions. A positive difference means TB outperforms BART. Each grid corresponds to a different prevalence setting.

S8. Real data application 3: Breast cancer tumour classification

For our third case study we use the Wisconsin breast cancer tumour dataset from the UCI repository (Dua and Graff, 2017). The dataset consists of $n = 699$ points, with covariates $\mathbf{x} \in \mathbb{R}^9$, which describe characteristics of the cell nuclei present in digitized images of a breast mass, and labels $y \in \{0, 1\}$. The class labels 0 and 1 correspond to 'benign' and 'malignant' cancers, respectively. To validate the results of the simulation in Section 3.3 in the main manuscript we artificially contaminate the dataset. More precisely, we use 70% of the data for training, which is corrupted by flipping the labels of 49 class 1 datapoints to 0 (10% contamination). In clinical practice, such data contamination may arise due to the manual nature of breast cancer detection and classification. Breast cancer detection is commonly performed through medical imaging modalities by one or more experts (usually pathologists) (Murtaza *and others*, 2019). The procedure is time-consuming and dependent on the professional experience and domain knowledge of the pathologists, thus making it prone to errors. This is highlighted by the significant inter- and intra-variability between pathologists (Warfield *and others*, 2008; Li *and others*, 2009; Hong *and others*, 2012). We use 20% of the training data as design and the rest as development set. We assume that missing a malignant cancer is more severe than misdiagnosing a benign as malignant, and so we focus on target thresholds $t < 0.5$, which correspond to a larger weight placed on false negatives vs false positives. Figure S4 presents the difference in NB for various t values over 5 splits of the training data into design and development. We see tailoring outperforms standard regression for most target thresholds.

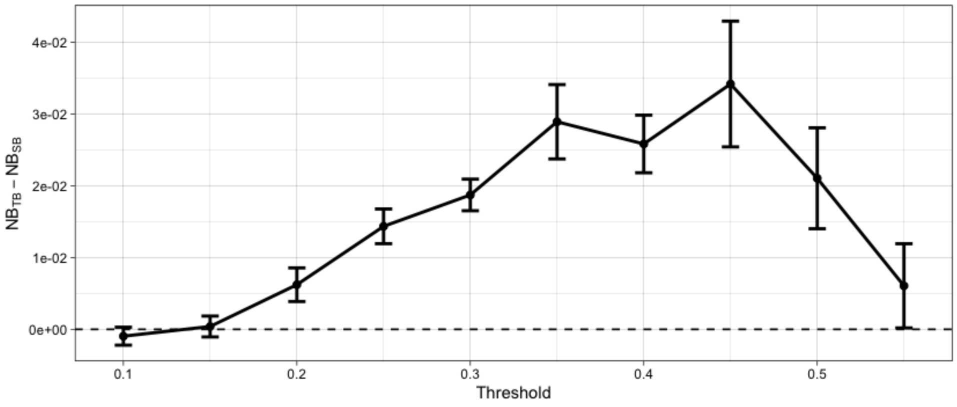

Fig. S4: Difference in Net Benefit for various t values evaluated on the test set. Error bars correspond to one standard error of the difference (see caption of Figure 7 main manuscript for details).

We further investigate the effect of tailoring on individual parameter values. Figure S5 shows the highest posterior density (HPD) regions under SB and TB for $t = 0.3$ and 0.5. As in the other case studies, under tailoring the regions are generally wider and are centred on different values. For instance, under $t = 0.3$ all posteriors are shifted towards more positives values. The only two exceptions are the coefficients of clump thickness, cell shape and mitosis which are pulled towards zero. Similar conclusions, but less pronounced are seen under $t = 0.5$. This again indicates that the relative importance of different features changes when using our tailored modelling approach.

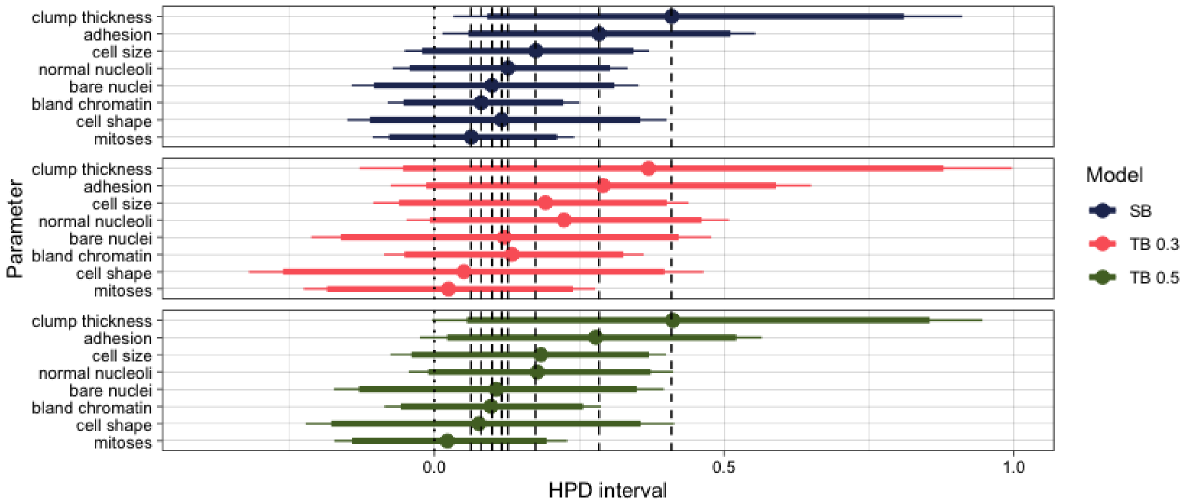

Fig. S5: Highest posterior density (HPD) regions for the parameters. Dots represent medians, and thick and thin lines represent 90 and the 95% of the HPD regions, respectively. The dashed vertical lines pass through the posterior median values of the SB parameters.

S9. Discussion Topics and Implementation Considerations

The method presented in this paper relies on the construction of the datapoint-specific weights (see (2.7) in the main manuscript). Here we discuss each element in turn.

S9.1 *On choosing* λ

We have opted to use cross-validation to choose λ. An open question is how to choose the range of λ values to consider. Our proposal is to consider values of the form $\lambda \in \{0, \ldots, m\}$. When $\lambda = 0$, the model reduces to standard logistic regression, a sensible choice for the lower limit. To choose the upper limit, m, note that as λ increases the rate with which the datapoints are downweighted increases exponentially (Figure S6). This in turn decreases the effective number of datapoints that are used when training the model. We call this the effective sample size for tailoring, ESS_T. Formally, we define ESS_T as

$$ESS_T = \sum_{i=1}^{n} w_i.$$

Under standard modelling, $ESS_T = n$, since $w_i = 1$, $\forall i$. Under tailoring $ESS_T \leqslant n$, which is why tailoring results in wider posteriors. This is demonstrated in Figure 1 of the main manuscript. In addition, Figure S7 shows the precision (as measured by the width) of the HPD credible intervals produced by each model under the simulation setting in Section 3.2 of the main manuscript. The figure suggests that the width of the credible intervals increases under tailoring compared to standard modelling. This is expected due to the downweighting of the likelihood contributions.

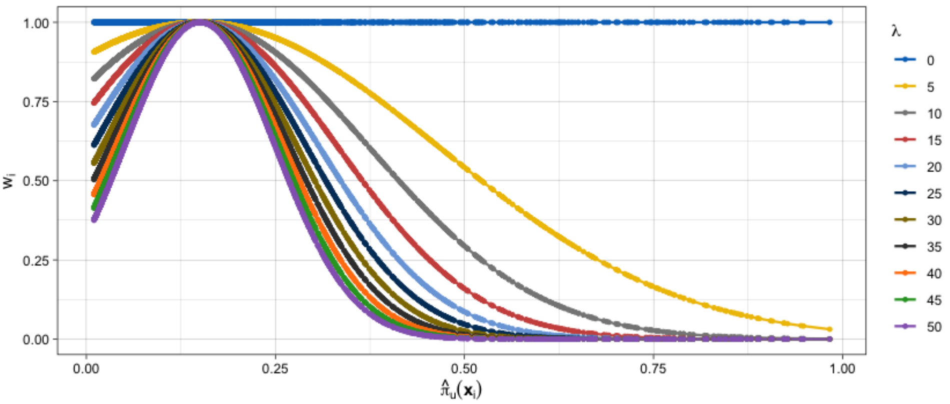

Fig. S6: Distribution of weights, w_i, against $\hat{\pi}_u(\mathbf{x}_i)$ for breast cancer prognostication case study (Section 4.1 main manuscript) for $t = 0.15$

As a result, we can use the ESS_T as a guide to choose m. Figure S8 shows $\frac{ESS_T}{n}$ for various λ values and target thresholds for the breast cancer prognostication case study (Section 4.1 main manuscript). Based on a target threshold we can choose m so the ESS_T does not drop below a pre-specified threshold. Importantly, this plot can be produced before fitting the model since we only need estimates of $\pi_u(\mathbf{x}_i)$.

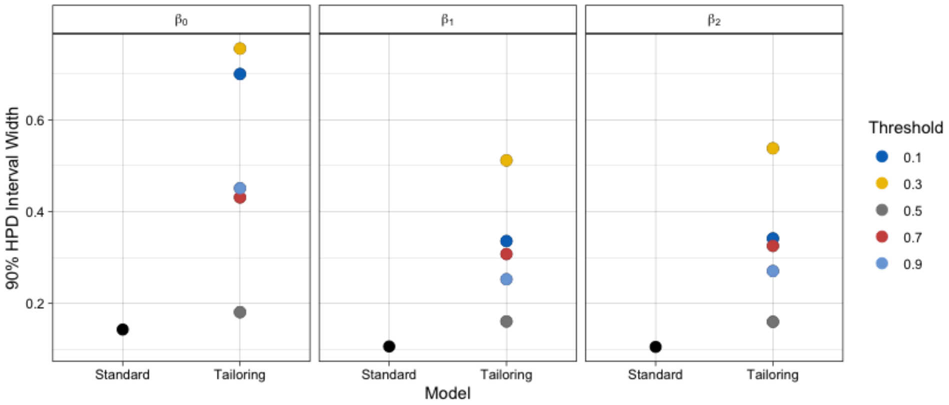

Fig. S7: 90% HPD Interval width for each parameter as a function of the model.

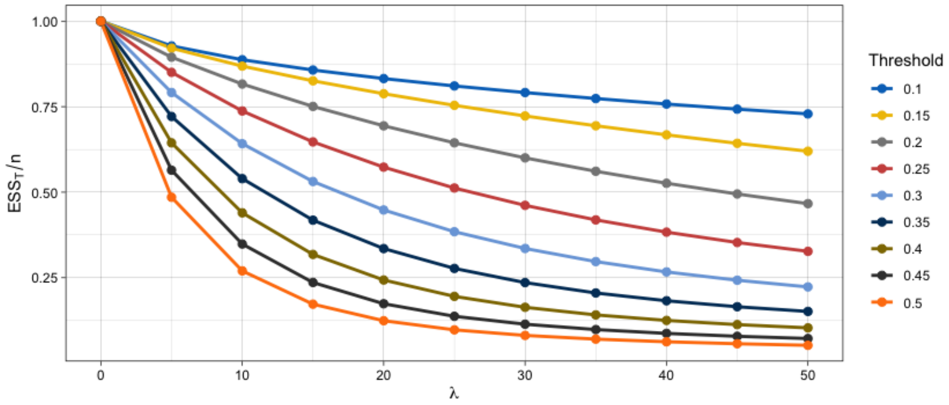

Fig. S8: $\frac{ESS_T}{n}$ for various λ values per target threshold.

In a similar fashion, we can have an indication whether TB will outperform SB before fitting the model. This can be achieved by plotting NB as λ increases (Figure S9). If NB remains stable or decreases as λ increases (Figure S9, $t = 0.5$ orange points) then TB will probably not offer any performance improvement compared to SB (see Figure 7 main text, $t = 0.5$). This is because, as discussed above, when $\lambda = 0$ TB reduces to SB (i.e., all weights are equal to one).

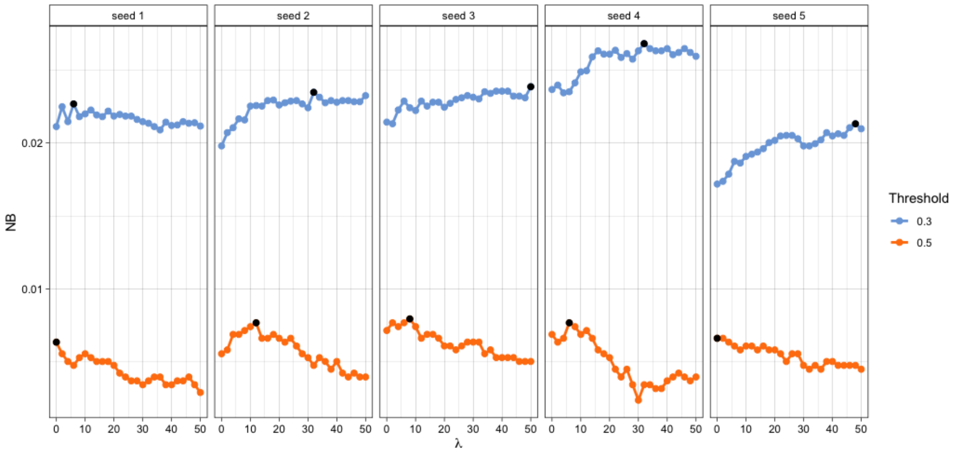

Fig. S9: Average 5-fold CV estimate of Net Benefit (NB) for the breast cancer prognostication dataset. Black points correspond to the chosen lambda values, λ^* (defined in Section S3).

S9.2 *On calibration*

Accurate estimation of $\pi_u(\mathbf{x}_i)$ at the first step of our framework is important for the construction of the weights. Ideally, we would like the estimated probabilities, $\hat{\pi}_u(\mathbf{x}_i)$ to be well calibrated. Calibration refers to the degree of agreement between observed and estimated probabilities. Probabilities are well calibrated if, for every 100 patients given a risk of x%, close to x have the event.

We use the breast cancer prognostication case study (Section 4.1 main manuscript) to investigate the effect of miscalibration on the model performance. First, we assess the calibration of $\hat{\pi}_u(\mathbf{x}_i)$. Figure S10a presents a graphical evaluation of calibration. It is based on loess-based smoothing method (Austin and Steyerberg, 2014) where the estimated (i.e., $\hat{\pi}_u(\mathbf{x}_i)$) and observed probabilities (from the development data) are plotted against each other; good models are close to the 45-degree line. We see the probabilities are well calibrated for the lower risks, and tend to be underestimated for the higher risks. To explore sensitivity of the tailored model to the accuracy of the step 1 probabilities, we deliberately perturbed $\hat{\pi}_u(\mathbf{x}_i)$ generating four miscalibration types: (1) overestimation; (2) underestimation, when probabilities are systematically overestimated or underestimated, respectively; (3) overfitting, when small probabilities are underestimated whereas large ones are overestimated; (4) underfitting, when small probabilities are overestimated whereas large ones are underestimated. We further allowed for two degrees of miscalibration (mild and severe) for each type giving us a total of eight scenarios (Figure S10b).

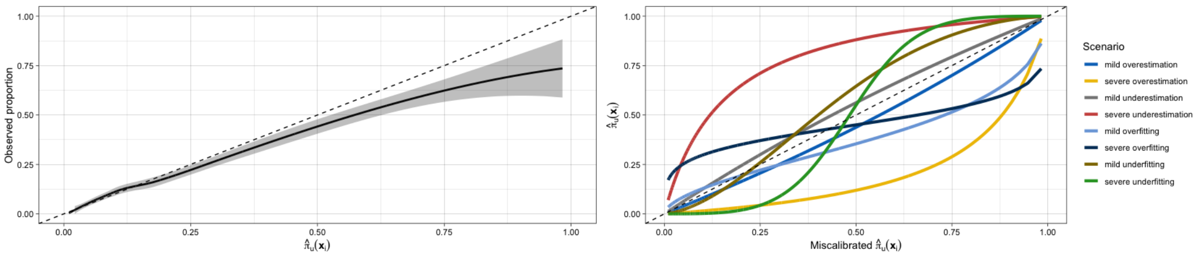

(a) (b)

Fig. S10: (a) Calibration plot of $\hat{\pi}_u(\mathbf{x}_i)$ on the train data using loess smoother. The 45-degree line represents the perfect calibration. (b) Illustrations of different miscalibration scenarios. The y axis shows $\hat{\pi}_u(\mathbf{x}_i)$ and the x axis the miscalibrated $\hat{\pi}(\mathbf{x}_i)$ used in model fitting.

Figure S11 shows the difference in NB between the original tailored (calibrated) model and the tailored miscalibrated ones for the different scenarios. Comparing across miscalibration types we see that the decline in performance depends on the type of miscalibration, with overfitting and underfittng more robust than over- and underestimation. Comparing within each type we note a drop in performance from mild to severe degrees, especially for over- and underestimation.

These results show that the model performance (in terms of NB) depends on the type of miscalibration and is robust to mild miscalibration. In practice, the calibration of the estimated probabilities can be readily evaluated graphically as done here or using statistical tests (Austin and Steyerberg, 2014). If the results show poor calibration we recommend re-calibrating $\hat{\pi}_u(\mathbf{x}_i)$ before calculating the datapoint-specific weights (Steyerberg *and others*, 2004; Janssen *and others*, 2008).

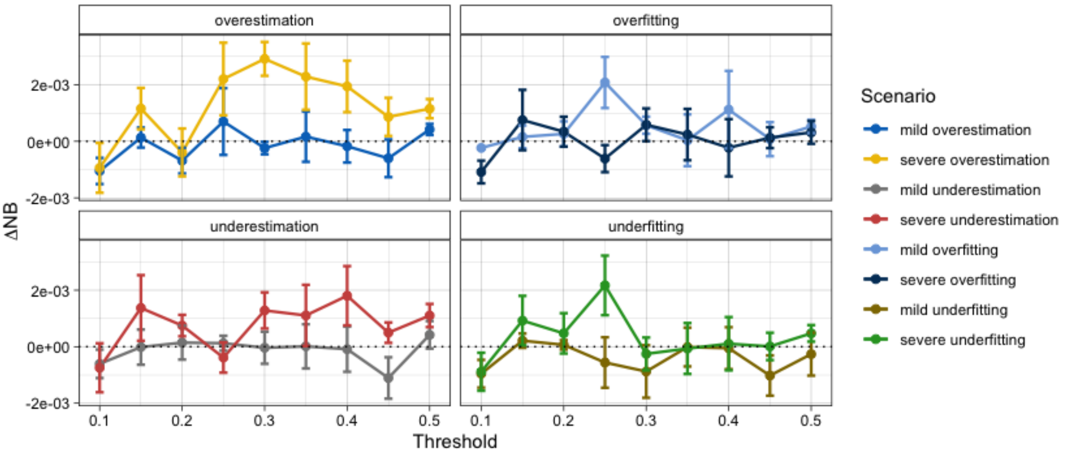

Fig. S11: Difference in NB, ΔNB (on the test set) between original, calibrated TB and miscalibrated TB under different scenarios. A positive difference means the calibrated TB outperforms the miscalibrated one.

S9.3 *On the weighting function*

In Section 2.3 of the main manuscript we defined the weights using the squared distance function, h. Here we investigate the sensitivity of the framework to the choice of the distance function. We choose the family of ϵ-insensitive functions (Vapnik, 1998), which is defined as

$$h(\pi_u(\mathbf{x}), t) = |\pi_u(\mathbf{x}) - t|_\epsilon$$

where we denote

$$|\pi_u(\mathbf{x}) - t|_\epsilon = \begin{cases} 0 & \text{if } |\pi_u(\mathbf{x}) - t| \leqslant \epsilon \\ |\pi_u(\mathbf{x}) - t| - \epsilon & \text{otherwise} \end{cases} \tag{S6}$$

The ϵ-insensitivity arises from the fact that the function value is equal to 0 if the discrepancy between the predicted probability $\pi_u(\mathbf{x})$ and the target threshold t is less than ϵ. In other words, we do not care about the distance as long as it is less than ϵ, but will not accept any deviation larger than this. As a result, observations with predicted probability within ϵ of the target threshold will not be downweighted. For $\epsilon = 0$ we recover the absolute distance, which is the objective function in median regression (Bassett Jr and Koenker, 1978). Both the squared distance and the family of ϵ-insensitive functions are symmetric, i.e., they downweight equally observations based only on their distance from the target threshold, not taking into account the direction. This is a reasonable requirement for our weighting function. Figure S12 presents the results for various ϵ values for the breast cancer case study (Section 4.1 main manuscript). The conclusions are qualitatively unchanged when compared within different ϵ values and between ϵ-insensitive functions and the squared distance (first panel in Figure S12). Hence, we conclude that for this dataset the results are also robust to the choice of the weighting function.

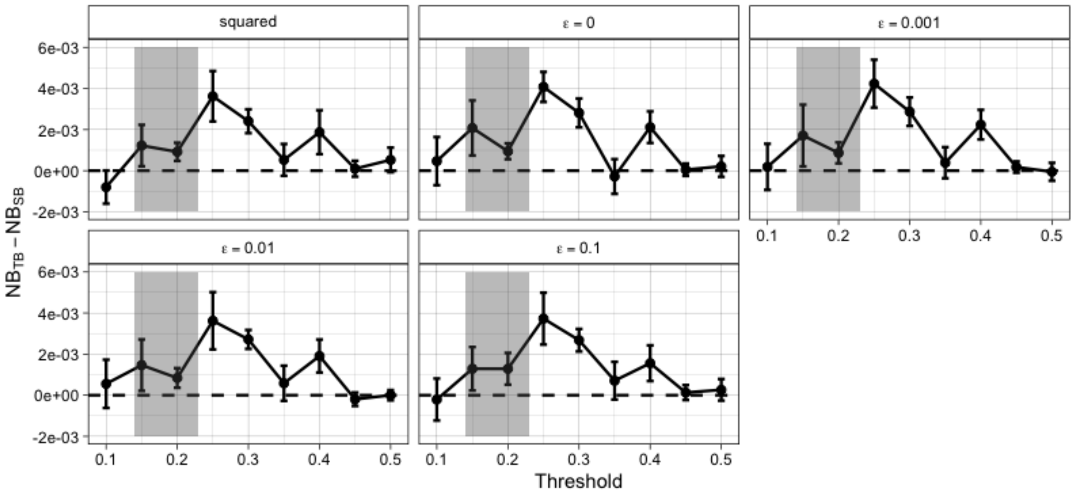

Fig. S12: Difference in NB (breast cancer prognostication case study) between TB and SB under the squared distance and ϵ-insensitive functions for various ϵ values. Note the first panel corresponds to Figure 7 in the main manuscript.

References

Austin, Peter C and Steyerberg, Ewout W. (2014). Graphical assessment of internal and external calibration of logistic regression models by using loess smoothers. *Statistics in Medicine* **33**(3), 517–535.

Bassett Jr, Gilbert and Koenker, Roger. (1978). Asymptotic theory of least absolute error regression. *Journal of the American Statistical Association* **73**(363), 618–622.

Brooks, Steve, Gelman, Andrew, Jones, Galin and Meng, Xiao-Li. (2011). *Handbook of Markov Chain Monte Carlo*. CRC press.

Dua, Dheeru and Graff, Casey. (2017). UCI machine learning repository.

Hong, Theodore S, Tomé, Wolfgang A and Harari, Paul M. (2012). Heterogeneity in head and neck IMRT target design and clinical practice. *Radiotherapy and Oncology* **103**(1), 92–98.

Janssen, KJM, Moons, KGM, Kalkman, CJ, Grobbee, DE and Vergouwe, Y. (2008). Updating methods improved the performance of a clinical prediction model in new patients. *Journal of Clinical Epidemiology* **61**(1), 76–86.

Li, X Allen, Tai, An, Arthur, Douglas W, Buchholz, Thomas A, Macdonald, Shannon, Marks, Lawrence B, Moran, Jean M, Pierce, Lori J, Rabinovitch, Rachel, Taghian, Alphonse *and others*. (2009). Variability of target and normal structure delineation for breast cancer radiotherapy: an RTOG multi-institutional and multiobserver study. *International Journal of Radiation Oncology* Biology* Physics* **73**(3), 944–951.

Linero, Antonio R and Yang, Yun. (2018). Bayesian regression tree ensembles that adapt to smoothness and sparsity. *Journal of the Royal Statistical Society: Series B* **80**(5), 1087–1110.

Murtaza, Ghulam, Shuib, Liyana, Wahab, Ainuddin Wahid Abdul, Mujtaba, Ghulam, Nweke, Henry Friday, Al-garadi, Mohammed Ali, Zulfiqar, Fariha, Raza, Ghulam and Azmi, Nor Aniza. (2019). Deep learning-based breast cancer classification through medical imaging modalities: state of the art and research challenges. *Artificial Intelligence Review*, 1–66.

Sparapani, Rodney, Spanbauer, Charles and McCulloch, Robert. (2021). Nonparametric machine learning and efficient computation with Bayesian additive regression trees: The BART R package. *Journal of Statistical Software* **97**(1), 1–66.

Steyerberg, Ewout W, Borsboom, Gerard JJM, van Houwelingen, Hans C, Eijkemans, Marinus JC and Habbema, J Dik F. (2004). Validation and updating of predictive logistic regression models: a study on sample size and shrinkage. *Statistics in Medicine* **23**(16), 2567–2586.

Vapnik, Vladimir. (1998). *Statistical Learning Theory*. Wiley.

Walker, Stephen and Hjort, Nils Lid. (2001). On Bayesian Consistency. *Journal of the Royal Statistical Society: Series B* **63**(4), 811–821.

Warfield, Simon K, Zou, Kelly H and Wells, William M. (2008). Validation of image segmentation by estimating rater bias and variance. *Philosophical Transactions of the Royal Society A: Mathematical, Physical and Engineering Sciences* **366**(1874), 2361–2375.
